# Epistatic Association Mapping for Alkaline and Salinity Tolerance Traits in the Soybean Germination Stage

**DOI:** 10.1371/journal.pone.0084750

**Published:** 2014-01-08

**Authors:** Wen-Jie Zhang, Yuan Niu, Su-Hong Bu, Meng Li, Jian-Ying Feng, Jin Zhang, Sheng-Xian Yang, Medrine Mmayi Odinga, Shi-Ping Wei, Xiao-Feng Liu, Yuan-Ming Zhang

**Affiliations:** 1 Section on Statistical Genomics, State Key Laboratory of Crop Genetics and Germplasm Enhancement, Department of Crop Genetics and Breeding, Nanjing Agricultural University, Nanjing, Jiangsu, China; 2 Institute of Crop Research, Ningxia Academy of Agriculture and Forestry Sciences, Yinchuan, Ningxia, China; Virginia Tech, United States of America

## Abstract

Soil salinity and alkalinity are important abiotic components that frequently have critical effects on crop growth, productivity and quality. Developing soybean cultivars with high salt tolerance is recognized as an efficient way to maintain sustainable soybean production in a salt stress environment. However, the genetic mechanism of the tolerance must first be elucidated. In this study, 257 soybean cultivars with 135 SSR markers were used to perform epistatic association mapping for salt tolerance. Tolerance was evaluated by assessing the main root length (RL), the fresh and dry weights of roots (FWR and DWR), the biomass of seedlings (BS) and the length of hypocotyls (LH) of healthy seedlings after treatments with control, 100 mM NaCl or 10 mM Na_2_CO_3_ solutions for approximately one week under greenhouse conditions. A total of 83 QTL-by-environment (QE) interactions for salt tolerance index were detected: 24 for LR, 12 for FWR, 11 for DWR, 15 for LH and 21 for BS, as well as one epistatic QTL for FWR. Furthermore, 86 QE interactions for alkaline tolerance index were found: 17 for LR, 16 for FWR, 17 for DWR, 18 for LH and 18 for BS. A total of 77 QE interactions for the original trait indicator were detected: 17 for LR, 14 for FWR, 4 for DWR, 21 for LH and 21 for BS, as well as 3 epistatic QTL for BS. Small-effect QTL were frequently observed. Several soybean genes with homology to *Arabidopsis thaliana* and soybean salt tolerance genes were found in close proximity to the above QTL. Using the novel alleles of the QTL detected above, some elite parental combinations were designed, although these QTL need to be further confirmed. The above results provide a valuable foundation for fine mapping, cloning and molecular breeding by design for soybean alkaline and salt tolerance.

## Introduction

Soil salinity and alkalinity are important abiotic stresses that adversely affect crop productivity and quality [1]. Approximately 20% of irrigated agricultural land is adversely affected by salinity and alkalinity [2], and salt-affected agricultural areas are continuously increasing. The salinity threat to agriculture exists in more than 100 countries [3]. Salinity inhibits seed germination and seedling growth; reduces nodulation; causes severe leaf chlorosis, bleaching and necrosis; and decreases total biomass and seed yield [4]–[6]. In China, there are 6.7 million ha of saline irrigated land, and 52.5–61.0% of soybean production loss is due to alkaline and salinity stresses [7]. The development of soybean cultivars with high salt tolerance is recognized as an efficient way to maintain sustainable soybean production in a salt stress environment [8]–[10]. However, the genetic architecture of the tolerance must first be elucidated. Therefore, the importance of alkaline and salt tolerance necessitates association mapping for these traits in soybean.

During the past decade, many attempts have been made to dissect the genetic mechanisms of alkaline and salt tolerances in *Arabidopsis* [11], [12], rice [13], [14] and tomato [15], with the greatest progress achieved in *Arabidopsis* and rice. First, studies showed that this type of tolerance is controlled by polygenes [12], . Then, many quantitative trait loci (QTL) for the tolerance were identified [11], [13], [17], [18]. Finally, a number of candidate genes and candidate pathways for the evolution of salinity tolerance have been reported [19] (http://www.arabidopsis.org/), e.g., the Na^+^/H^+^ ion antiporter gene *AtNHX1* [20], the salt overly sensitive pathway gene *SOS1–SOS3* [21]–[23], the K^+^/Na^+^ homeostasis genes *SKC1* [24] and *Saltol* [25], the stomatal aperture control gene *DST* [26] and the ABA signaling or synthesis gene *RAS1* [27]. However, very little is known about the genetics of alkaline and salt tolerances in soybean.

Although there have been some classic inheritance studies of salt tolerance in soybean [28], [29], the molecular inheritance of this tolerance needs to be addressed. Lee et al. [30] identified one major QTL for salt tolerance, which was associated with markers sat_091, satt237 and satt339 on linkage group N. Based on the assumption that this QTL was identical to the *Ncl* locus identified by Abel [28], this locus was further confirmed in F_2_ and recombinant inbred lines (RIL) [10], [31]. Additional loci have been reported; e.g., Chen et al. [16] detected 11 QTL in RIL; Lee et al. [32] found a different single dominant gene in F_2_; and Tuyen et al. [33] mapped a major QTL on linkage group D2 in F_2_ and F_6_. In addition, Cho et al. [34] detected two markers, satt285 and satt079, that were significantly associated with foliar TRG accumulation, which is correlated with NaCl stress in soybean. It should be noted that all the above results in soybean were obtained from biparental populations. However, biparental population mapping strategies have several drawbacks [35]. For example, if the two parental lines do not segregate at a particular QTL, the QTL cannot be detected regardless of how many offspring are sampled in the mapping population. To overcome such shortcomings, association mapping strategies are recommended [36], [37], and many such studies have been conducted [38]–[47]. However, very little is known about the detection of both QTL-by-environment (QE) and QTL-by-QTL (QQ) interactions.

The objective of this study was to mine novel QTL for alkaline and salt tolerances in soybean using epistatic association mapping (EAM) [36]. Elite alleles from the detected QTL were used to design parental combinations for cultivar improvement.

## Results

### Evaluation of phenotypic values for STI and ATI

Alkaline and salt tolerances were measured in the LR, FWR, DWR, BS and LH of 257 soybean cultivars in 2009 and 2010, and the mean values, standard deviations, ranges, skewness and kurtosis were calculated (Tables 1 and S1). All the traits exhibited continuous distribution, and most showed a normal distribution. It should be noted that 2 germplasm accessions, Fengzitianandou and Baiqiu 1, were found to be highly resistant to salt; furthermore, 8 germplasm accessions, Linanbayuebai, Shengxiantiangengdou, Zunyizongzidou, Beichuanwuyanwo, Fengzitianandou, Guangxidalidou, Hedou 6 and Jidou 13, were highly resistant to alkaline condition.

**Table 1:** Phenotypic variation in alkaline and salt tolerance indices measured in 257 soybean cultivars in 2009 and 2010.

Year	Indicator	Trait	Mean	Std Dev	Minimum	Maximum	Skewness	Kurtosis
2009	STI	LR	44.58	12.53	−2.22	67.15	−0.88	0.68
		LH	51.03	14.67	−0.26	82.48	−0.50	0.16
		FWR	63.92	13.51	9.81	95.71	−0.84	1.58
		DWR	51.56	15.67	−8.00	87.37	−0.53	0.31
		BS	47.22	9.64	21.52	74.89	−0.18	−0.03
	ATI	LR	32.24	18.38	−19.36	89.95	−0.04	0.87
		LH	19.24	16.86	−42.05	72.88	−0.12	1.13
		FWR	62.67	16.96	−8.96	95.06	−1.52	4.08
		DWR	49.14	18.98	−30.00	91.04	−0.85	2.13
		BS	34.03	11.68	−10.41	64.11	−0.82	1.79
2010	STI	LR	45.26	9.23	1.70	69.00	−0.72	1.99
		LH	46.80	15.25	5.47	79.24	−0.34	−0.20
		FWR	50.48	12.55	0.31	82.69	−0.43	1.03
		DWR	32.54	18.18	−28.57	71.43	−0.70	0.80
		BS	40.46	10.71	−30.65	66.94	−1.12	7.47
	ATI	LR	21.71	16.48	−20.57	66.01	0.11	−0.08
		LH	11.54	12.01	−34.91	42.86	−0.46	0.73
		FWR	40.98	22.26	−17.29	82.16	−0.52	−0.38
		DWR	30.90	21.49	−23.53	78.57	−0.22	−0.52
		BS	23.52	13.74	−24.13	50.46	−0.47	−0.05

STI: salt tolerance index; ATI: alkaline tolerance index; LR: Length of main root; FWR: fresh weights of roots; DWR: dry weights of roots; BS: biomass of seedlings; LH: length of hypocotyls.

The analysis of variance showed significant differences among all the cultivars for all the traits (Table 2), indicating that genetic variation exists among all the cultivars. Most of the other studied factors were also significant (Table 2), suggesting that treatment, year and interactions should be considered in joint association mapping.

**Table 2:** Analysis of variance in the length of the main root (LR), fresh and dry weights of roots (FWR and DWR), biomass of seedlings (BS) and length of hypocotyls (LH).

Source of variation	DF	Length of main root		Length of hypocotyls		Fresh weight of roots		Dry weight of roots		Biomass of seedlings	
		MS	F	MS	F	MS	F	MS	F	MS	F
Year	1	3268.810	2689.26**	287.580	341.04**	3.625	1977.39**	0.006	4.17*	12.547	1190.54**
Treat	2	9080.705	7470.72**	5287.965	6271.05**	13.870	7565.39**	0.044	30.98**	84.024	7972.70**
Year × Treat	2	179.925	148.02**	17.630	20.91**	0.206	112.27**	0.010	6.60**	0.793	75.23**
Variety	256	34.002	27.97**	11.707	13.88**	0.070	38.12^**^	0.002	1.24*	0.749	71.04**
Year × Variety	236	15.650	12.88**	4.400	5.22**	0.019	10.54**	0.002	1.15	0.130	12.36**
Treat × Variety	512	6.760	5.56**	3.724	4.42**	0.012	6.72**	0.001	1.00	0.060	5.69**
Year × Treat × Variety	461	4.579	3.77**	2.079	2.47**	0.008	4.38**	0.002	1.06	0.031	2.92**
Residual	1447	1.216		0.843		0.002		0.001		0.011	

*and **: significance at the 0.05 and 0.01 levels, respectively. DF: degree of freedom; MS: mean square.

### Mapping QTL for STI traits

A total of 83 QE interactions (24 for LR-STI, 12 for FWR-STI, 11 for DWR-STI, 15 for LH-STI and 21 for BS-STI) and one epistatic QTL of FWR-STI were detected using EAM implemented with an empirical Bayes algorithm. Among these QTL, 19 were confirmed using an enriched compressed mixed linear model (E-cMLM) method. Most of the detected QTL showed small effects on these traits, except for one LR-STI QTL associated with satt022 and one FWR-STI epistatic QTL between markers satt656 and sat_256. A summary of all the detected QTL, including the marker name, linkage group, position, variance, *r* ^2^ value and QTL type, is presented in Table 3. If two linked QTL were separated by less than 5 cM, they were considered a single QTL.

**Table 3:** Association mapping for alkaline and salt tolerance indices using epistasis association mapping.

Trait	Epistatic association mapping						Similar results		Trait	Epistatic association mapping						Similar results	
	Marker	Linkage group	Position (cM)	LOD	Var	*r* ^2^ (%)	E-cMLM	Previous study		Marker	Linkage group	Position (cM)	LOD	Var	*r* ^2^ (%)	E-cMLM	Previous study
LR-STI	sat_267	A1	78.44	6.51	1.81	1.51			LR-STI	satt215	J	44.08	7.55	0.63	0.52		Chen et al (2008)
	satt200	A1	92.88	4.21	0.49	0.41				satt247-satt417	K	43.95–46.2	3.08–12.64	0.29–3.20	0.24–2.66	satt102 (10)	Chen et al (2008)
	satt390	A2	9.14	11.09	0.07	0.06				satt463-satt245	M	50.09–53.54	3.03–4.38	0.83–1.57	0.69–1.30		Chen et al (2008)
	satt632	A2	51.5	4.64	0.76	0.63				satt323	M	60.04	4.48	0.39	0.32		Chen et al (2008)
	satt509	B1	32.5	12.36	0.28	0.24		Chen et al (2008)		satt022	N	102.05	15.56	8.94	7.43		Lee et al (2004); Chen et al (2008)
	satt453	B1	123.95	3.09	2.12	1.76			LR-ATI	satt329	A2	110.94	4.95	0.74	0.22		
	satt565	C1	0.00	3.23	2.94	2.45				satt070	B2	72.8	3.62	4.46	1.35		
	satt289	C2	112.34	4.19	0.70	0.59	√(10§)	Cho et al (2002)		satt277	C2	107.58	3.10	7.06	2.13		
	sat_254	D1b	46.91	6.73	2.09	1.74		Chen et al (2008)		satt514	D2	85.69	3.22	2.41	0.73		Tuyen et al (2010)
	satt226	D2	85.15	4.84	2.79	2.32	sat_354☆(09), satt615(10)			satt615-sat_354	D2	91.2–93.7	3.29–4.13	2.07–9.08	0.63–2.75		Tuyen et al (2010)
	satt615-sat_354	D2	91.2–93.7	4.59–7.29	1.05–5.94	0.87–4.94	satt615(10), sat_354(09)			satt659	F	26.7	6.34	9.40	2.84	√(09)	
	satt045	E	46.65	4.39	1.74	1.45	√(09)			satt160	F	33.18	11.43	8.71	2.64	√(09)	
	sat_381	E	64.18	3.05	0.48	0.40	satt045(09)			satt132	J	39.18	3.48	7.76	2.35	√(10)	
	AW186493	F	21.04	3.41	0.42	0.35				satt431	J	78.57	3.85	1.35	0.41	√(09)	
	satt160	F	33.18	4.90	2.55	2.12				satt247	K	43.95	6.06	6.02	1.82		
	satt309	G	4.53	4.92	2.21	1.83		Cho et al (2002)		satt238	L	19.93	3.53	3.94	1.19	√(10)	
	AF162283	G	87.94	5.41	0.47	0.39		Chen et al (2008)		satt652	L	30.87	5.63	0.86	0.26	√(09)	
	satt222	H	68.08	3.85	0.46	0.39				satt245	M	53.54	3.92	3.99	1.21	sat_256 (09)	
	sat_228	J	23.91	3.24	0.48	0.40		Chen et al (2008)		satt683	N	34.52	4.66	5.44	1.64	√(09, 10)	
LR-ATI	sat_280	N	43.45	5.78	9.71	2.94	satt683(09, 10)		FWR-ATI	satt142	H	86.48	3.86	3.43	0.67	√(10)	
	satt237	N	74.98	4.48	1.28	0.39				satt215	J	44.08	5.37	2.40	0.47	satt414 (10)	
	satt022	N	102.05	10.29	13.89	4.20				satt417	K	46.2	10.28	7.33	1.44		
FWR-STI	satt289	C2	112.34	7.28	1.13	0.53		Cho et al (2002)		sct_190	K	77.37	16.51	1.85	0.36		
	satt296	D1b	52.61	7.08	0.99	0.46		Chen et al (2008)		satt238	L	19.93	4.61	3.13	0.62	√(09)	
	sat_354	D2	93.7	5.99	6.65	3.10	satt488 (10)			satt527	L	70.36	7.19	1.56	0.31		
	satt672	D2	114.97	3.20	0.36	0.17				satt463	M	50.09	4.49	15.36	3.02	√(09)	
	satt649	F	5.36	4.57	3.71	1.73				satt094	O	56.58	4.55	7.44	1.46		
	satt659	F	26.7	3.28	3.11	1.45			DWR -STI	satt045	E	46.65	3.85	2.41	0.64		
	satt352	G	50.52	3.20	4.04	1.89	satt138 (09)	Chen et al (2008)		AW186493	F	21.04	3.72	0.50	0.13		
	satt440	I	112.69	3.49	4.06	1.90				satt160	F	33.18	5.56	7.73	2.05		
	satt463	M	50.09	4.46	1.41	0.66		Chen et al (2008)		satt688	G	12.54	4.20	1.19	0.32		Cho et al (2002)
	sat_256	M	74.52	11.55	6.12	2.86		Chen et al (2008)		satt440	I	112.69	4.20	2.78	0.74		
	satt022	N	102.05	9.30	4.20	1.96		Li et al (2004); Chen et al (2008)		satt441	K	46.2	6.17	1.78	0.47		Chen et al (2008)
	satt262	O	57.02	3.92	0.44	0.21				sct_190	K	77.37	3.23	1.51	0.40	√(09)	
	satt656×sat_256	F×M	135.11×74.52	3.40	33.83	15.80	satt656 (10)	Chen et al (2008)		satt220	M	56.29	4.39	4.19	1.11		Chen et al (2008)
FWR-ATI	sat_344	A1	19.37	3.66	15.00	2.95				satt250	M	107.69	3.61	2.39	0.63		Chen et al (2008)
	satt632	A2	51.5	3.38	5.11	1.00				sat_266	N	47.27	3.08	6.85	1.81	satt631(09)	
	satt577	B2	6.05	3.09	4.53	0.89	sat_342 (10)			satt022	N	102.05	3.08	6.02	1.59		Lee et al (2004); Chen et al (2008)
	AW277661	C1	74.79	4.93	5.61	1.10			DWR -ATI	satt382	A1	26.42	9.90	14.04	2.85		
	sat_354	D2	93.7	7.23	15.26	3.00	sat_365 (10)	Tuyen et al (2010)		satt509-sat_261	B1	32.5–32.95	3.61–5.89	1.39–1.92	0.28–0.39		
	satt452	E	45.09	3.09	2.77	0.54				satt070	B2	72.8	5.12	9.98	2.03		
	satt160	F	33.18	6.28	21.18	4.16				staga001	C2	119.84	5.35	3.82	0.78		
	satt352	G	50.52	4.70	5.43	1.07											
DWR-ATI	satt307	C2	121.26	4.28	0.47	0.10			LH-STI	satt309	G	4.53	15.41	7.39	3.25		Cho et al(2002)
	satt282	D1b	76.09	3.81	6.55	1.33				sat_372	G	107.75	3.46	4.58	2.01		
	sat_354	D2	93.7	6.19	13.17	2.67		Tuyen et al (2010)		satt132	J	39.18	4.34	4.95	2.18		Chen et al (2008)
	satt263	E	45.4	4.10	4.93	1.00				satt683	N	34.52	3.34	1.66	0.73		
	satt149	F	18.12	3.81	1.26	0.26				sat_280-sat_266	N	43.45–47.27	3.78–4.11	2.26–5.84	0.99–2.56		
	satt142	H	86.48	7.14	1.88	0.38	√(2010)			satt262	O	57.02	4.89	3.61	1.59		
	satt132	J	39.18	3.08	6.92	1.40	satt215(09,10)		LH-ATI	satt382	A1	26.42	3.59	8.02	3.53		
	satt417	K	46.2	4.11	4.51	0.92				sat_342	B2	20.3	3.31	7.76	3.42		
	sct_190-satt260	K	77.37–80.12	4.10–3.64	1.99–2.39	0.40–0.49				staga001	C2	119.84	11.30	8.28	3.65		
	satt463	M	50.09	4.23	15.36	3.12	satt323(10)			satt357	C2	151.91	3.40	1.04	0.46		
	satt220-satt626	M	56.29–58.59	3.49–3.60	1.12–5.19	0.23–1.05	satt323 (10)			sat_254	D1b	46.91	4.90	1.88	0.83	√(09)	
	sat_256	M	74.52	3.97	10.68	2.17	satt323 (10)			satt672	D2	114.97	3.90	0.59	0.26	satt413(09)	
	satt592	O	100.37	3.31	9.23	1.87	sat_274 (10)			satt452	E	45.09	5.51	4.65	2.05	satt685(09); satt263(10)	
LH-STI	satt200	A1	92.88	5.52	0.53	0.23				AW186493	F	21.04	5.16	0.68	0.30	satt160 (10)	
	satt632	A2	51.5	4.88	4.06	1.79	aw132402(09)			satt656	F	135.11	6.18	3.39	1.49		
	aw132402	A2	67.86	12.53	3.20	1.41	√(09)			satt309	G	4.53	4.37	5.20	2.29		
	sat_355	B2	66.23	4.58	1.83	0.81		Chen et al (2008)		satt688	G	12.54	4.86	0.15	0.07		
	staga001	C2	119.84	8.20	2.64	1.16	sat_252 (09)	Cho et al (2002)		sat_372	G	107.75	3.04	5.81	2.56		
	sat_160	D1a	104.27	5.15	3.68	1.61				satt302	H	81.04	7.24	0.15	0.07		
	satt411	E	12.92	8.64	1.33	0.58				satt354	I	46.22	4.84	2.26	0.99	satt419 (09)	
	satt045	E	46.65	13.30	5.73	2.51	√(09)			sat_419	I	98.11	3.46	3.68	1.62		
satt160	F	33.18	6.15	10.25	4.50				satt440	I	112.69	7.93	2.18	0.96			
										satt260	K	80.12	4.25	1.59	0.70		
LH-ATI	satt255	N	76.48	3.60	0.24	0.10			BS-STI	satt237	N	74.98	5.84	1.45	1.26		Lee et al (2004);Chen et al (2008)
BS-STI	satt382	A1	26.42	3.50	2.79	2.42				satt022	N	102.05	4.21	3.05	2.66		Lee et al (2004);Chen et al (2008)
	aw132402	A2	67.86	3.56	1.06	0.92			BS-ATI	sat_344	A1	19.37	4.72	3.88	2.04		
	satt453	B1	123.95	4.13	1.03	0.90				satt382	A1	26.42	5.72	5.51	2.90		
	satt070	B2	72.8	5.28	2.11	1.84	√(09)	Chen et al (2008)		satt577	B2	6.05	4.84	3.19	1.68	sat_342 (10)	
	satt534	B2	87.58	4.21	3.08	2.68	satt070(09)			AW277661	C1	74.79	5.63	2.69	1.42		
	AW277661	C1	74.79	4.05	0.81	0.71				satt422	C2	44.66	4.22	0.11	0.06		
	satt640	C2	30.46	3.20	0.37	0.32				staga001	C2	119.84	10.08	4.52	2.38	√(09)	
	satt422	C2	44.66	3.73	0.73	0.63				satt669	D2	67.7	4.25	1.14	0.60	satt226(10)	Tuyen et al (2010)
	satt289	C2	112.34	3.93	0.62	0.54		Cho et al (2002)		sat_354	D2	93.7	6.24	5.66	2.98	satt226(10)	Tuyen et al (2010)
	satt296	D1b	52.61	4.02	0.40	0.35		Chen et al (2008)		satt263	E	45.4	5.13	1.71	0.90		
	sat_354	D2	93.7	5.39	3.82	3.32	√(09)			satt160	F	33.18	9.66	9.42	4.96		
	satt452-satt045	E	45.09–46.65	3.50–5.63	0.49–1.36	0.42–1.18				satt138	G	55.99	6.76	1.18	0.62		
	satt659	F	26.7	5.81	1.56	1.35				sat_372	G	107.75	4.10	6.20	3.27		
	satt160	F	33.18	8.59	4.09	3.56				satt417	K	46.2	9.64	2.71	1.43		
	satt309	G	4.53	6.91	2.24	1.95		Cho et al (2002)		sct_190	K	77.37	3.81	0.84	0.44		
	satt270	I	50.11	6.90	0.88	0.77				satt626	M	58.59	3.06	0.87	0.46		
	sat_228	J	23.91	4.02	0.23	0.20		Chen et al (2008)		sat_256	M	74.52	8.91	6.80	3.58		
	sat_256	M	74.52	5.28	3.80	3.30	satt245(09)	Chen et al (2008)		satt631	N	26.13	3.01	1.57	0.83		
	satt250	M	107.69	3.51	0.44	0.39		Chen et al (2008)		satt022	N	102.05	3.37	5.70	3.00		

: the same associated marker detected by different approaches;

: year, 09:2009; 10: 2010;

: marker in similar result column is linked to the associated marker in this study; LR: Length of main root; FWR: Fresh weight of root; DWR: Dried weight of root; LH; Length of hypocotyl; BS: Biomass.

A total of 24 LR-STI QTL, with total phenotypic variance explained (PVE) values of 0.06–7.43%, were identified and mapped to linkage groups A1, A2, B1, C1, C2, D1b, E-H and J-N. Among these QTL, 6 were further identified by E-cMLM. It should be noted that the QTL associated with satt022 had a PVE greater than 5%. Furthermore, the two QTL associated with markers satt615 and sat_354 should be considered a single QTL because the genetic distance between the two markers is 2.5 cM. In addition, the QTL associated with markers satt226, satt615 and sat_354 were simultaneously identified in 2009 and 2010 using E-cMLM.

A total of 13 FWR-STI QE, with 0.17–3.10% PVE, were identified and mapped to linkage groups C2, D1b, E, G, I and M-O. One epistatic QTL with 15.80% PVE was identified between markers satt656 and sat_256, and sat_256 was also found to exhibit environmental interaction. Among these QTL, 3 were also identified using E-cMLM.

A total of 11 DWR-STI QE, with 0.13-2.05% PVE, were found and mapped to linkage groups E-G, I, K, M and N. Of these QTL, 3 were also identified using E-cMLM.

A total of 15 LH-STI QE, with 0.23–4.50% PVE, were identified and mapped to linkage groups A1, A2, B2, C2, D1a, E-G, J, N and O. Of these QTL, 4 were also identified using E-cMLM. Several closely linked QTL, e.g., sat_280 and sat_266 (3.82 cM), were considered a single QTL.

A total of 21 BS-STI QE, with 0.20–3.56% PVE, were identified and mapped to linkage groups A1-G, I, J, M and N. Of these QTL, 4 were also identified using E-cMLM. Closely linked QTL, e.g., satt452 and satt045 (1.56 cM), were considered a single QTL.

### Mapping QTL for ATI traits

A total of 86 QE interactions (17 for LR-ATI, 16 for FWR-ATI, 17 for DWR-ATI, 18 for LH-ATI and 18 for BS-ATI) were identified using EAM implemented with an empirical Bayes algorithm. Among these QTL, 29 were confirmed using an E-cMLM method. Most of the detected QTL showed small effects on these traits. A summary of all the detected QTL is presented in Table 3.

A total of 17 LR-ATI QTL, with 0.41–4.20% PVE, were detected and mapped to linkage groups A2, B2, C2, D2, F and J-N. Of these QTL, 8 were also identified using E-cMLM. It should be noted that several markers were associated with both LR-ATI and LR-STI, i.e., satt615-sat_354, satt160, satt247, satt245 and satt022. Fourteen closely linked marker pairs were found to be associated with both LR-STI and LR-ATI, e.g., satt289 (LR-STI) and satt277 (LR-ATI). In addition, using E-cMLM, two markers, satt683 and sat_280, were found to be associated with LR-ATI in 2009 and 2010.

A total of 16 FWR-ATI QE, with 0.31–4.16% PVE, were detected and mapped to linkage groups A1, A2, B2, C1, D2, E–H, J–M and O. Of these QTL, 6 were also identified using E-cMLM. It should be noted that several markers were associated with both FWR-ATI and FWR-STI, i.e., sat_354, satt352 and satt463. Three closely linked marker pairs were associated with both FWR-STI and FWR-ATI, e.g., satt262 (FWR-STI) and satt094 (FWR-ATI).

A total of 17 DWR-ATI QE, with 0.10–3.12% PVE, were identified and mapped to linkage groups A1, B1, B2, C2, D1b, D2, E, F, H, J, K, M and O. Of these QTL, 6 were also detected using E-cMLM. Several closely linked QTL were considered a single QTL, e.g., satt509 and sat_261 (0.45 cM). It should be noted that two markers were associated with both DWR-ATI and DWR-STI, i.e., sct_190 and satt220, and nine closely linked marker pairs were associated with both DWR-STI and DWR-ATI, e.g., satt441 (DWR-STI) and satt417 (DWR-ATI). In addition, using E-cMLM, marker satt132 was found to be associated with DWR-ATI in 2009 and 2010.

A total of 18 LH-ATI QE, with 0.10–3.12% PVE, were found and mapped to linkage groups A1, B2, C2, D1b, D2, E-I, K and N. Of these QTL, 5 were also identified using E-cMLM. It should be noted that three markers were associated with both LH-ATI and LH-STI, i.e., staga001, satt309 and sat_372. Three closely linked marker pairs were associated with both LH-STI and LH-ATI, e.g., satt045 (LH-STI) and satt452 (LH-ATI). In addition, using E-cMLM, marker satt452 was found to be associated with LH-ATI in 2009 and 2010.

A total of 18 BS-ATI QE, with 0.06–4.96% PVE, were detected and mapped to linkage groups A1, B2, C1, C2, D2-G, K, M and N. Of these QTL, 4 were also identified using E-cMLM. Seven markers were associated with both BS-ATI and BS-STI, i.e., satt382, AW277661, satt422, sat_354, satt160, sat_256 and satt022. Five closely linked marker pairs were associated with both BS-STI and BS-ATI, e.g., satt289 (BS-STI) and staga001 (BS-ATI).

In summary, the above mapping results produced three particularly noteworthy observations. First, almost all the detected QTL were small (1–5%). Second, almost all the detected QTL were found to exhibit environmental interactions. Finally, some markers were found to be associated with the STI and ATI indicators of multiple traits. For example, marker sat_354 was associated with LR-ATI, LR-STI, FWR-ATI, FWR-STI, BS-ATI and BS-STI, and markers satt160 and satt022 were associated with LR-STI, LR-ATI, BS-STI and BS-ATI. These data indicate that correlations exist among the above five traits (Table 2).

### Mapping QTL from original traits

Original trait observations and marker information were used to perform EAM. A total of 78 QE (17 for LR, 14 for FWR, 4 for DWR, 21 for LH and 21 for BS) and 3 epistatic QTL for BS were detected and are listed in Table 4. Among these QTL, 74 were also identified using E-cMLM. Most of the detected QTL showed small effects on these traits, except for two epistatic QTL: sat_390 × sat_254 and satt244 × sat_160.

**Table 4:** Association mapping for alkaline and salt tolerance traits using epistasis association mapping and an enriched compressed mixed linear model (E-cMLM).

Trait	Epistatic association mapping						Similar result		
	Marker	Linkage group	Position (cM)	LOD	Var	*r* ^2^(%)	E-cMLM	Jointly E-cMLM	Previous study
LR	satt289	C2	112.34	24.49	0.03	0.18	sat_252☆(09CK§,09AL)	staga001(M×Y)	Cho et al(2002)
	sat_252	C2	127	4.69	0.10	0.73	√(09CK, 09AL)	√(M×Y)	Cho et al(2002)
	satt580	D1a	62.36	6.55	0.02	0.18		satt077(M×Y)	
	satt296	D1b	52.61	4.70	0.03	0.19	√(09CK)	satt282(M×Y)	Chen et al (2008)
	satt459	D1b	118.62	5.37	0.08	0.56	satt274(10AL)	√(M×Y)	
	satt672	D2	114.97	4.32	0.03	0.21	satt413(10CK,10SA)	satt413(M×Y)	
	satt263	E	45.4	3.33	0.06	0.45	satt452(09AL), sat_381(10AL)	√(M×Y)	
	satt149	F	18.12	4.31	0.03	0.25	√(10CK), satt659(09CK), BE806387(10AL)	AW186493(M×Y)	
	satt222	H	68.08	3.76	0.07	0.48	satt142(09SA)	√(M×Y)	
	sat_224	J	75.12	3.15	0.13	0.98	√(09CK), satt431(09AL)		
	satt244	J	65.04	3.79	0.32	2.38	sat_224(09CK), satt431(09AL)	sat_165(M×Y)	Chen et al (2008)
	sct_190	K	77.37	7.81	0.04	0.30		√(M×Y)	
	satt166	L	66.51	27.34	0.04	0.28			
	satt683	N	34.52	7.86	0.11	0.81	sat_266(10AL)	satt631(M×Y)	
	satt234	N	84.59	3.47	0.01	0.07	satt237(10SA)	satt255(M×Y)	Li et al (2004);Chen et al (2008)
	satt022	N	102.05	3.86	0.31	2.31		√(M×Y)	Li et al (2004);Chen et al (2008)
	satt331	O	93.37	6.07	0.04	0.29	satt592(09SA)	√(M×Y)	
FWR	sat_267	A1	78.44	3.40	0.00	0.86	satt200(09CK,10CK)	√(M)	
	satt390	A2	9.14	3.14	0.00	0.25		√(M,M×T)	
	satt509	B1	32.5	6.39	0.00	0.37			Chen et al (2008)
	satt289	C2	112.34	18.14	0.00	0.27	satt277(09SA,09AL,10SA)	√(M)	Cho et al (2002)
	sat_252	C2	127	4.17	0.00	0.99	satt277(09SA, 09AL,10SA)	√(M)	Cho et al (2002)
	satt672	D2	114.97	8.78	0.00	0.23	satt256(09CK,09SA,10CK)	√(M)	
	sat_381	E	64.18	6.12	0.00	0.78		√(M)	
	satt269	F	11.37	6.21	0.00	0.21	BE806387(09CK,09AL),satt149(10SA)	satt149(M)	
	sat_390	F	1.79	3.40	0.00	1.06	BE806387(09CK,09AL),satt149(10SA)	√(M)	
	satt215	J	44.08	3.81	0.00	0.35	√(09SA),satt132(10AL)	satt244(M)	Chen et al (2008)
	sct_190	K	77.37	40.07	0.00	0.19	satt260(10SA)	√(M)	
	satt238	L	19.93	3.86	0.00	0.30		satt652(M)	
	satt463	M	50.09	5.31	0.00	0.87	√(09AL),satt626(09CK),satt245(09SA)	√(M,M×Y)	Chen et al (2008)
	satt234	N	84.59	87.87	0.00	0.06	satt022(09CK),satt237(09SA)	satt237(M)	Li et al (2004);Chen et al (2008)
DWR	satt453	B1	123.95	3.83	0.00	4.38	√(10SA)		
	satt669	D2	67.7	3.11	0.00	0.13			
	satt302	H	81.04	3.33	0.00	0.32			
	sct_190	K	77.37	10.32	0.00	0.08			
LH	satt453	B1	123.95	5.65	0.05	0.75	√(09SA)	√(M)	
	sat_355	B2	66.23	3.78	0.01	0.22	satt534(10SA)	√(M)	Chen et al (2008)
	satt289	C2	112.34	24.06	0.02	0.25	sat_252(09CK),staga001(09SA,10SA,10AL),satt202(10CK)	satt277(M)	Cho et al (2002)
	satt254	D1a	56.43	3.49	0.03	0.42	satt580(09SA)	AW781285(M)	
	satt580	D1a	62.36	11.47	0.00	0.01	satt580(09-NaCl)	AW781285(M)	
	satt296	D1b	52.61	10.20	0.03	0.48	√(09AL,10SA,10AL),sat_254(09CK)	√(M)	Chen et al (2008)
	sat_254	D1b	46.91	4.36	0.06	1.00	√(09CK),satt296(09AL,10SA,10AL)	satt296(M)	Chen et al (2008)
	satt669	D2	67.7	3.55	0.02	0.33	satt226(10CK)	satt226(M)	Tuyen et al (2010)
	sat_354	D2	93.7	4.75	0.12	2.00	√(09SA),satt514(10CK)	√(M,M×Y)	Tuyen et al (2010)
	sat_381	E	64.18	7.56	0.03	0.46	√(09AL),satt045(09SA),satt263(10AL)	√(M)	
	satt149	F	18.12	16.35	0.00	0.07	BE806387(09CK,09AL,10CK,10AL),AW186493(09SA),satt659(10SA)	BE806387(M)	
	satt160	F	33.18	3.74	0.11	1.82	BE806387(09CK,09AL,10CK),AW186493(09SA),satt659(10SA,10AL)	√(M)	
	satt309	G	4.53	4.25	0.10	1.55	satt688(10CK)	√(M,M×Y)	Cho et al(2002)
	satt222	H	68.08	4.03	0.02	0.36		√(M,M×Y)	
	satt354	I	46.22	3.73	0.06	0.98	√(10CK,10AL),satt270(09CK,09AL)	√(M,M×Y)	
	sat_228	J	23.91	4.41	0.02	0.39	sat_165(09AL,10SA,10AL)	sat_165(M)	Chen et al (2008)
	satt441	K	46.2	4.61	0.03	0.42		√(M)	Chen et al (2008)
	satt166	L	66.51	5.46	0.01	0.17	satt527(10SA)		
	satt683	N	34.52	18.58	0.03	0.43		√(M)	
	satt234	N	84.59	85.46	0.00	0.07		satt237(M,M×Y)	Li et al (2004);Chen et al (2008)
	sat_266	N	47.27	5.49	0.06	0.97		√(M)	
BS	satt390	A2	9.14	5.67	0.00	0.22	√(09SA,09AL)	√(M)	
	satt233	A2	100.08	6.10	0.00	0.55	√(09CK,10SA,10AL)	√(M,M×Y)	
	satt453	B1	123.95	4.13	0.00	0.46		√(M)	
	satt565	C1	0	5.65	0.00	0.53			
	sat_252	C2	127	3.84	0.00	0.74	satt277(09SA,09AL)	√(M)	Cho et al (2002)
	satt580	D1a	62.36	3.41	0.00	0.21	AW781285(10CK)	√(M×Y)	
	satt274	D1b	116.34	9.04	0.00	0.58	√(09CK,09AL,10AL)	√(M)	
	satt256	D2	124.3	3.28	0.00	0.51	√(09CK,09SA,09AL)	satt672(M)	
	satt413-satt672	D2	113.61–114.97	5.91–13.60	0.00	0.27–0.41	satt256(09CK,09SA,09AL)	√(M)	
	sat_381	E	64.18	5.37	0.00	0.74		√(M)	
	satt269	F	11.37	6.44	0.00	0.27	BE806387(09CK,09AL)	√(M)	
	satt656	F	135.11	4.82	0.00	0.95		√(M)	Chen et al (2008)
	satt659×sat_354	F×D2	26.7×93.7	3.04	0.01	3.29	BE806387(09CK,09AL),sat_354(09SA)	satt659(M),sat_362(M)	Tuyen et al (2010)
	sat_390×sat_254	F×D1b	1.79×46.91	3.23	0.01	9.00	BE806387(09CK,09AL),sat_254(09CK)	sat_390(M),satt296(M)	
	satt222	H	68.08	7.70	0.00	0.29	√(09SA,09AL)	√(M)	
	satt132	J	39.18	4.79	0.00	0.46	satt215(09AL)	√(M)	Chen et al (2008)
	satt244	J	65.04	4.18	0.00	1.60	satt215(09AL)	√(M)	Chen et al (2008)
	satt244×sat_160	J×D1a	65.04×104.27	3.15	0.02	11.41	satt215(09AL)	satt244(M),sat_160(M)	
	sct_190	K	77.37	42.52	0.00	0.11	√(09CK,09AL),satt260(10CK,10SA)	√(M)	
	satt238	L	19.93	4.23	0.00	0.18		satt652(M)	
	sat_391	M	1.02	5.57	0.00	1.36	√(10CK)	√(M)	
	satt234	N	84.59	20.49	0.00	0.08	satt022(09CK,09AL)	√(M)	Li et al (2004);Chen et al (2008)
	satt022	N	102.05	3.97	0.00	1.42	√(09CK,09AL)	√(M)	Li et al (2004);Chen et al (2008)
	satt331	O	93.37	5.54	0.00	0.18	sat_274(09AL)	√(M)	

: the same association marker detected by different approach;

: year, i.e., 2009, and 2010; SA: salt; AL: alkaline.

: marker in similar result column is linked to the associated marker in this study; M: main-effect QTL; Y: year; T: treatment. LR: Length of main root; FWR: Fresh weight of root; DWR: Dried weight of root; LH; Length of hypocotyl; BS: Biomass.

A total of 17 QE interactions for LR, with 0.07–2.38% PVE, were detected and mapped to linkage groups C2, D1a-F, H, J–L, N and O. Of these QTL, 16 were also identified using E-cMLM. Note that seven markers, satt289, sat_252, satt672, satt263, satt149, sat_224 and satt244, were found to be associated with LR in more than two environments. When all the LR datasets were jointly analyzed by E-cMLM, 15 QTL-by-year interactions were detected, which was consistent with the identification of 15 QE interactions using EAM.

A total of 14 FWR QE interactions, with 0.06–1.06% PVE, were identified and mapped to linkage groups A1-B1, C2, D2-F and J-N. Of these QTL, 13 were also identified using E-cMLM. Note that nine QTL were found to be associated with LR in more than two environments. When all the FWR datasets were jointly analyzed using E-cMLM, 13 main-effect QTL, one QTL-by-treatment interaction and QTL-by-year interaction were detected, which was consistent with the identification of 13 QE interactions using EAM.

Four DWR QE interactions, with 0.08-4.38% PVE, were identified and mapped to linkage groups B1, D2, H and K. Of these QTL, one was also identified using E-cMLM.

A total of 21 LH QE interactions, with 0.07-2.00% PVE, were identified and mapped to linkage groups B1, B2, C2-L and N. All of these QTL were also identified using E-cMLM. Note that nine QTL were found to be associated with LH in more than two environments. When all the LH datasets were jointly analyzed using E-cMLM, 20 main-effect QTL and five QTL-by-year interactions were detected. Among these QTL, twenty were found to be consistent with the QE interactions for LH index identified using EAM.

A total of 21 QE and 3 QQ interactions for BS, with 0.08–11.41% PVE, were detected and mapped to linkage groups A2, B1, C2-F, H and J-O. Of these QTL, 23 were also identified using E-cMLM. Several closely linked QTL were considered a single QTL, e.g., satt413 and satt672 (1.36 cM), and thirteen QTL were found to be associated with BS in more than two environments. When all the BS datasets were jointly analyzed using E-cMLM, 26 main-effect QTL and two QTL-by-year interactions were detected. Among these QTL, 23- were found to be consistent with the QE and QQ interactions for BS identified using EAM.

A comparison of the QTL for ATI and STI indicators revealed 56 common QTL: 14 for LR, 11 for FWR, 3 for DWR, 15 for LH and 13 for BS. For example, markers satt289, satt222 and satt022 were associated with both LR and LR-STI, and markers satt683 and satt022 were associated with both LR and LR-ATI. Among these markers, marker satt022 is common; in other words, marker satt022 was associated with LR, LR-STI and LR-ATI.

### Predictions for novel parental combinations

The best way to improve a trait is to pyramid all the desirable elite alleles into one cultivar, if possible. By maximizing the number of elite alleles using a Monte Carlo simulation experiment, the ideal novel cultivar combination can be designed. Using this method, 21 elite alleles of 27 QTL for LR-STI were pyramided by combining cultivars Guangxibayuehuang, 0804, Shangqiu 832012, Qingyuanxiaoqingdou, Daheidou, Ganjiangnan, Shuangliuliuyuehuang, Baiqiu 1, Xu 0701 and Wenfeng 5.

## Discussion

The mapping results from this study are reliable for three reasons. First, 19 QTL for STI, 29 QTL for ATI, and 74 QTL for original traits, which were detected using EAM, were confirmed by E-cMLM (Tables 3 and 4). Furthermore, 36 QTL for STI (11 for LR-STI, 7 for FWR-STI, 5 for DWR-STI, 4 for LH-STI and 9 for BS-STI), 6 QTL for ATI (2 for LR-ATI, 1 for FWR-ATI, 1 for DWR-ATI and 2 for BS-ATI) and 29 QTL for original traits (6 for LR, 6 for FWR, 10 for LH and 7 for BS) found in this study have also been identified by other researchers (Tables 3 and 4). For example, one major QTL on linkage group N plays an essential role in enhancing soybean salt tolerance in different genetic backgrounds [10], [28], [30], and the present study also confirmed several associations with salt tolerance, e.g., satt237 for BS-STI and satt234 for LR, LH, FWR and BS. Chen et al. [16] identified 11 QTL significantly associated with salt tolerance, and Cho et al. [34] detected 15 QTL on linkage groups B2, C2, D2, G, J and K for foliar TRG accumulation, which is postulated to function as a compatible solute and/or osmoprotectant under adverse NaCl stress conditions. These 26 QTL were all confirmed in this study. Finally, several salt tolerance genes from *Arabidopsis thaliana* and soybean (http://www.ncbi.nlm.nih.gov/gene?term=salt%20tolerance) were found to be located in close proximity to tolerance-associated markers in this study.

The potential candidate genes for salt tolerance are summarized in Tables S2 and S3. Among these candidate genes, four soybean genes, Glyma13g41980, Glyma15g03400, Glyma17g37430 and Glyma17g35340, which have homologs in *Arabidopsis*, were associated with five markers examined in this study, with physical distances of 135.76–820.04 kb. Six soybean salt tolerance genes, *LOC100808889*, *LOC100807827*, *LOC100800981*, *LOC100795117*, *LOC100814727* and *LOC100797515*, were closely linked to seven markers analyzed in this study, with physical distances of 135.41–619.74 kb. Although these consistent results were observed, one might raise doubts about our conclusions because we did not consider cultivar relatedness. In our opinion, the inclusion of all the main and epistatic QTL in the genetic model reduced the importance of controlling for genetic background.

Tuyen et al. [33] reported that tolerance to NaCl may not always accompany tolerance to alkaline stress. Their evidence was based on a discrepancy between the QTL for alkaline tolerance, located between markers satt669 and sat_300 on linkage group D2, and the QTL for saline tolerance, located on linkage group N, in wild soybean JWS156-1. However, the opposite phenomenon was observed in the present study. For example, satt237 on linkage group N was associated with both LR-ATI and BS-STI; satt615 on linkage group D2 was associated with both LR-STI and LR-ATI; and sat_354 on linkage group D2 was associated with LR-STI, LR-ATI, FWR-STI, FWR-ATI, BS-STI and BS-ATI. Similar results for other linkage groups were also found in this study. More importantly, the cultivar Fengzitianandou was found to be highly resistant to NaCl and alkaline stresses. In addition, tolerance to salt and alkaline stresses is related to tolerance to Al in soybean.

Sharma et al. [48] identified two major QTL for tolerance to Al. These two QTL were also detected in the present study and were located in the marker intervals satt160-satt252 (F) and satt202-satt371 (C2), respectively. Note that satt160 was simultaneously associated with LR-STI, LR-ATI, FWR-ATI, DWR-STI, LH-STI, BS-STI, BS-ATI and LH. Bianchi-Hall et al. [49] identified six QTL associated with tolerance to Al stress. Among these QTL, *Al tol1-1*, *Al tol1-3*, *Al tol1-5* and *Al tol1-6* were found to be located in close proximity to the associated markers in this study. For example, satt329 (associated with LR-ATI) and satt233 (associated with BS) on linkage group A2 were close to *Al tol1-1*; satt509 (associated with LR-STI, DWR-ATI and FWR) on linkage group B1 was close to *Al tol1-3*; satt215 (associated with LR-STI, FWR-ATI and FWR), satt431 (associated with LR-ATI), satt132 (associated with DWR-ATI, LH-STI and BS), satt244 (associated with LR and BS) and sat_224 (associated with LR) on linkage group J were close to *Al tol1-5*; and satt238 (associated with LR-ATI, FWR-ATI, FWR and BS) on linkage group L was linked to *Al tol1-6*. These results demonstrate that the mechanisms of tolerance to alkaline/salt and Al conditions are similar.

Most of the detected QTL showed small effects, except for four QTL: one main-effect QTL associated with satt022 and three epistatic QTL: satt656 × sat_256 for LR-STI, sat_390 × sat_254 for BS and satt244 × sat_160 for BS. The results of this study demonstrate that large differences in tolerance are caused not only by a few large-effect QTL but also by the cumulative effect of numerous small-effect QTL. This small-effect QTL phenomenon has been observed in *Arabidopsis* [50], rice [51] and maize [52], although the high PVE of the epistatic QTL is surprising because alkaline and salt tolerances in plants are derived from interactive molecular pathways [53]. In addition, almost all QTL exhibit significant environmental interactions, indicating that the genetic architecture across various salt and alkaline stresses is sensitive or variable.

Research into tolerance to NaCl and alkaline stresses usually employs two types of indicators: a salt and alkaline tolerance index and an original trait indicator. These two types of indicators were also analyzed in this study, producing complementary results: tolerance genes were found proximal to both types of associated markers.

The utilization of varieties with multiple resistance genes is an effective way to reduce the effects of adversity. As the numbers of both cloned genes and detected QTL increase, a Monte Carlo simulation experiment becomes a valuable means of parental combination prediction and selection strategy design [36], [54]. In this study, several parental combinations were predicted. Certain cultivars are encountered repeatedly in these combinations and may be used to improve multiple traits. For an STI indicator, e.g., Sudou 1 may be chosen for improving FWR and BS; Daheidou for LR and LH; Qingyuanxiaoheidou for LR, LH and BS; Qinyan 1 for DWR and BS; and Wuhuabayuehuang for DWR and BS. These predictions may be valuable for several reasons. First, two of the selected parents, Zhejiangsiyuebai (DWR-STI) and Caoqing (BS-STI), are on the list of 348 ancestors of 651 soybean cultivars released during 1923–1995 in China [55]. Youbian 31, Sudou 1, Fengjiao 66–12, Ludou 1, Wenfeng 5, and Jindou 2 are on the list of 171 varieties that were bred earlier and were derived from 519 soybean cultivars [55]. Second, some accessions exhibit beneficial agronomic traits and strong resistance to adversity, i.e., Sudou 1, Ludou 1, Kexi 8, Daheidou, Edou 2, Niumaohuang, Wenfeng 5, Dianbaiheidou and Baiqiu 1. Finally, some selected cultivars have been widely grown in certain areas, i.e., Sudou 1, Ludou 1 and Wenfeng 5. In addition, we should consider the sizes of novel alleles when predicting parental combinations. Thus, the tolerant variety Fengzitianandou may be included in a parental combination because of its large effect. Of course, a prerequisite for the above prediction is that all the above QTL will be further confirmed.

The phenomenon of QTL clusters has been reported in soybean [56] and cotton [57]. Previous work has indicated that numerous disease resistance loci are clustered in various regions of the soybean genome, e.g., on chromosomes D1b and F. This phenomenon was also evident in the current results. For example, QTL linked to markers satt226, satt615, sat_354 and satt514 on chromosome D2 were clustered in an 8.55 cM interval region. Three salt tolerance genes, Glyma17g37430, Glyma17g35340 and LOC100807827, were found to be located near the tolerance-associated QTL clusters of satt256, satt413 and satt672 (Tables S2 and S3). The phenomenon of QTL clusters may be used to explain trait correlation [57].

## Materials and Methods

### Plant materials and DNA marker analysis

**Figure 1:**
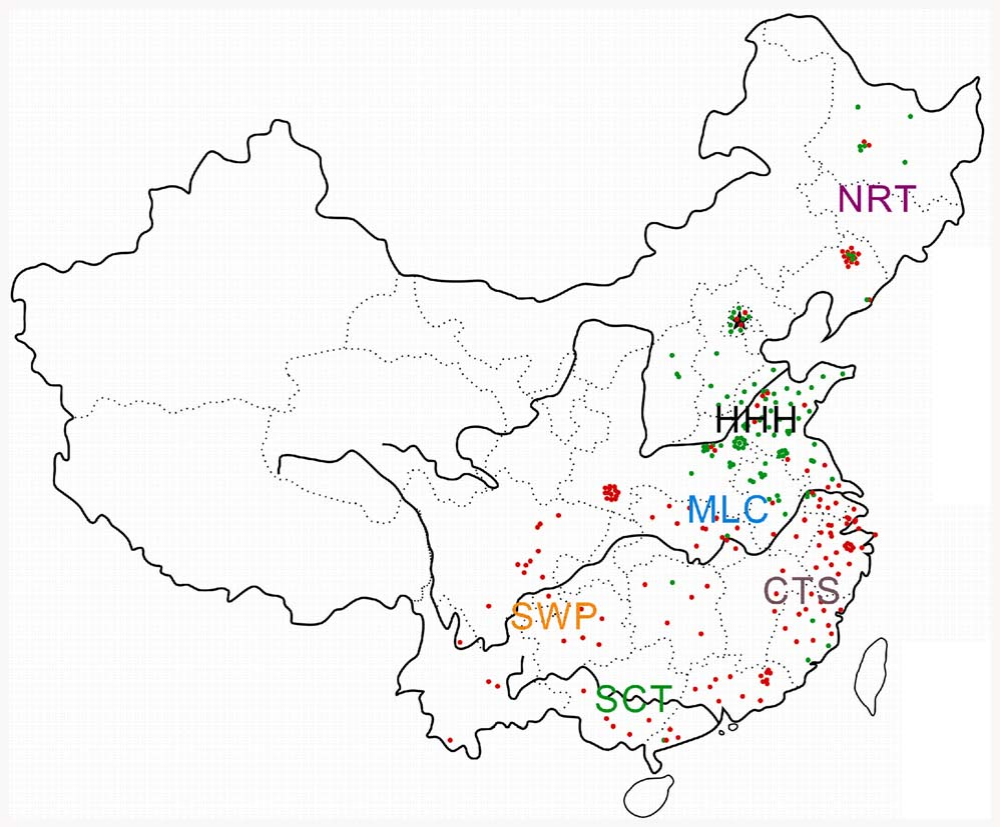
Distribution of 257 soybean cultivars.

All the 257 soybean cultivars were obtained, by stratified random sampling, from 6 geographic ecotypes in China (Fig. 1), were planted in three-row plots in a completely randomized design, and were evaluated at the Jiangpu experimental station at Nanjing Agricultural University in 2009 and 2010. The plots were 1.5 m wide and 2 m long. These seeds were used to conduct plastic container experiments.

Approximately 0.3 g of fresh leaves obtained from each cultivar in 2009 was used to extract genomic DNA using the cetyltrimethylammonium bromide method, as described by Lipp et al. [58]. To screen for polymorphisms among all the cultivars, PCR was performed with 135 simple sequence repeat (SSR) primer pairs. The primer sequences were obtained from the soybean database Soybase (http://www.ncbi.nlm.nih.gov). PCR was performed as described by Xu et al. [56].

### Evaluation of alkaline and salt tolerances

A salt-water flooding method [59] was used to evaluate the alkaline and salt tolerances of all the soybean cultivars. In brief, twelve soybean seeds for each cultivar were sown in a 30×20×15 cm plastic container with sand added to a height of 3.5×cm. The seeds were then treated with control (CK, pH: 7.0), 100×mM NaCl (pH: 7.0) and 10×mM Na_2_CO_3_ (pH: 11.1) solutions, with two replications each. A 350-ml aliquot of the appropriate solution for each treatment was applied to each plastic container filled with sand. Twelve soybean seeds for each treatment were grown in a growth chamber under white fluorescent light (600 µmol m^−2^ s^−1^; 14 h light/10 h dark) at 25±1°C. The length of the main root (LR), fresh and dry weights of roots (FWR and DWR), biomass of seedlings (BS) and length of hypocotyls (LH) of healthy seedlings from 5 plants in plastic containers under simulated alkaline and salt conditions were measured 7 days after sowing. The units used were centimeters for length and grams for weight. To measure the degree of salt and alkaline tolerances, the original trait observations may be transformed into salt and alkaline tolerance indices for each trait using the below equations:
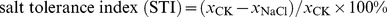


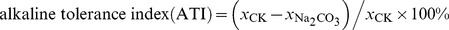
where 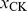,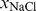 and 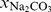 stand for the phenotypic values exhibited following control, saline and alkaline treatments, respectively.

### Population structure

**Figure 2:**
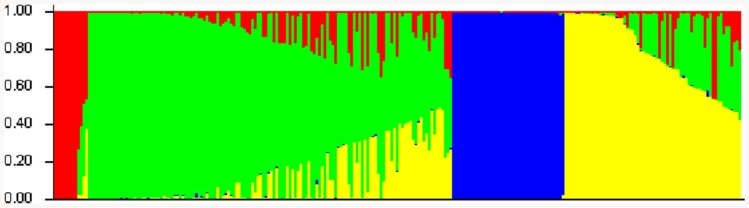
Plot of posterior probabilities (*y*-axis) against four subgroups on each cultivar (*x*-axis) using STRUCTURE software.

Population structure plays an important role in association mapping. To investigate the population structures of all the selected cultivars, the STRUCTURE program [60] and the approach of Evanno et al. [61] were employed. The number of subpopulations (*K*) was set from 2 to 10. The number of replicates for each *K* was 20, and the total average of the mean log-likelihood at a fixed value of *K* was used. Using the ad hoc statistic Δ*K*, which is based on the rate of change in the log-probability of data between successive *K* values, the Δ*K* value was much higher for the model parameter *K* = 4 than for other values of *K* (Fig. 2). By combining this high Δ*K* value with knowledge of the breeding history of these cultivars, a *K* value of 4 was chosen. The *Q* matrix was calculated based on information from 135 SSR markers and was incorporated into the association mapping.

### Epistatic association mapping

The EAM approach suggested by Lü et al. [36] was also used to analyze all the five-trait datasets in this study; for technical details, the reader is referred to the original study by Lü et al. [36]. All the above analyses were performed using the SAS program.

The enriched compression mixed linear model (E-cMLM) approach, suggested by Li [37] and expanded by Zhang et al. [62], was used to confirm the results of the epistatic association mapping. In this analysis, the dataset for each year and each treatment was analyzed, and all the datasets for all the two-year and three-treatment datasets were jointly analyzed.

## Supporting Information

Table S1 **Phenotypic variation in the length of the main root (LR), fresh and dry weights of roots (FWR and DWR), biomass of seedlings (BS) and length of hypocotyls (LH) among healthy seedlings measured in 257 soybean cultivars in 2009 and 2010.**
(DOC)

Table S2 **SSR markers located near soybean genes with homology to salt tolerance genes in** ***Arabidopsis thaliana.***
(DOC)

Table S3 **SSR markers located near salt tolerance genes in soybean.**
(DOC)
