# Supplementary material for: Epistatic Association Mapping for Alkaline and Salinity Tolerance Traits in the Soybean Germination Stage
Source: PLoS One. 2014 Jan 8;9(1):e84750. doi: 10.1371/journal.pone.0084750 (PMC3885605; doi:10.1371/journal.pone.0084750)
Supplement: Table S1 — Phenotypic variation in the length of the main root (LR), fresh and dry weights of roots (FWR and DWR), biomass of seedlings (BS) and length of hypocotyls (LH) among healthy seedlings measured in 257 soybean cultivars in 2009 and 2010. (DOC) [file pone.0084750.s001.doc]

**Table S1.** Phenotypic variation in the length of main root (LR), fresh and dry weights of roots (FWR and DWR), biomass of seedlings (BS) and length of hypocotyls (LH) among healthy seedlings measured in 257 soybean cultivars in 2009 and 2010

| **Year** | **Trait** | **Treatment** | **Mean** | **Std** | **Minimum** | **Maximum** | **Skewness** | **Kurtosis** |
| --- | --- | --- | --- | --- | --- | --- | --- | --- |
| 2009 | LR | CK | 12.21 | 3.12 | 4.9 | 21.21 | 0.33 | -0.36 |
|  |  | NaCl | 6.57 | 1.49 | 3.14 | 12.04 | 0.52 | 0.55 |
|  |  | Na_2_CO_3_ | 8.02 | 2.46 | 1.55 | 16.21 | 0.49 | 0.76 |
|  | LH | CK | 9.02 | 1.58 | 5.08 | 13.47 | 0.16 | 0.17 |
|  |  | NaCl | 4.34 | 1.31 | 1.85 | 8.7 | 0.49 | -0.02 |
|  |  | Na_2_CO_3_ | 7.26 | 1.96 | 2.11 | 24.61 | 2.99 | 24.81 |
|  | FWR | CK | 0.36 | 0.14 | 0.09 | 0.87 | 0.71 | 0.76 |
|  |  | NaCl | 0.13 | 0.07 | 0.01 | 0.46 | 1.1 | 1.59 |
|  |  | Na_2_CO_3_ | 0.13 | 0.07 | 0.03 | 0.42 | 1.82 | 3.95 |
|  | DWR | CK | 0.03 | 0.02 | 0.01 | 0.2 | 4.72 | 38.32 |
|  |  | NaCl | 0.02 | 0.01 | 0 | 0.08 | 2.91 | 13.62 |
|  |  | Na_2_CO_3_ | 0.01 | 0.01 | 0.01 | 0.04 | 1.38 | 2.09 |
|  | BS | CK | 1.29 | 0.36 | 0.47 | 2.65 | 0.55 | 0.91 |
|  |  | NaCl | 0.68 | 0.22 | 0.22 | 1.36 | 0.74 | 0.39 |
|  |  | Na_2_CO_3_ | 0.84 | 0.26 | 0.33 | 1.77 | 0.9 | 1.22 |
| 2010 | LR | CK | 14.24 | 2.7 | 6.86 | 22.07 | -0.08 | -0.16 |
|  |  | NaCl | 7.77 | 1.6 | 3.75 | 13.46 | 0.36 | 0.43 |
|  |  | Na_2_CO_3_ | 11.12 | 2.98 | 4.29 | 18.79 | 0.3 | -0.4 |
|  | LH | CK | 9.35 | 1.56 | 5.67 | 13.96 | 0.32 | 0.16 |
|  |  | NaCl | 4.9 | 1.34 | 2.05 | 9.14 | 0.56 | 0.2 |
|  |  | Na_2_CO_3_ | 8.21 | 1.39 | 4.41 | 12.72 | 0.12 | -0.12 |
|  | FWR | CK | 0.4 | 0.13 | 0.11 | 0.82 | 0.39 | 0.25 |
|  |  | NaCl | 0.2 | 0.08 | 0.04 | 0.41 | 0.31 | -0.46 |
|  |  | Na_2_CO_3_ | 0.23 | 0.12 | 0.06 | 0.69 | 1.19 | 1.66 |
|  | DWR | CK | 0.03 | 0.01 | 0.01 | 0.05 | 0.46 | 0.01 |
|  |  | NaCl | 0.02 | 0.01 | 0 | 0.05 | 0.45 | -0.05 |
|  |  | Na_2_CO_3_ | 0.02 | 0.06 | 0.01 | 1.01 | 15.07 | 232.3 |
|  | BS | CK | 1.35 | 0.37 | 0.49 | 2.44 | 0.11 | 0.11 |
|  |  | NaCl | 0.81 | 0.25 | 0.23 | 1.48 | 0.13 | -0.21 |
|  |  | Na_2_CO_3_ | 1.04 | 0.32 | 0.32 | 2.15 | 0.57 | 0.49 |
