# Supplementary material for: Epistatic Association Mapping for Alkaline and Salinity Tolerance Traits in the Soybean Germination Stage
Source: PLoS One. 2014 Jan 8;9(1):e84750. doi: 10.1371/journal.pone.0084750 (PMC3885605; doi:10.1371/journal.pone.0084750)
Supplement: Table S2 — SSR markers located near soybean genes with homology to salt tolerance genes in Arabidopsis thaliana. (DOC) [file pone.0084750.s002.doc]

***Table S2****. SSR markers located near soybean genes with homology to salt tolerance genes in Arabidopsis thaliana*

| **Salt tolerance genes in *Arabidopsis thaliana*** | |  | **Homologous genes in soybean** | | |  | **SSR markers that are around soybean homologous gene** | | | |
| --- | --- | --- | --- | --- | --- | --- | --- | --- | --- | --- |
| **Gene** | **Annotation** |  | **Gene** | **Chr** | **Position** |  | **Marker** | **Position** | **Distance to soybean gene (bp)** | **Trait** |
| AT1G06040 (STO) | DNA binding/ protein binding/ transcription factor/zinc ion binding |  | [Glyma13g41980](http://chibba.pgml.uga.edu/duplication/index/locus_app?lc=Glyma13g41980) | F | 42125437-42123426 |  | satt656 | 41884911-41885065 | 240.37 | BS |
|  |  |  | Glyma15g03400 | E | 2379542-2381511 |  | satt411 | 2517275-2517372 | 135.76 | LH-STI |
| AT1G75540 (STH2) | transcription factor/ zinc ion binding |  | [Glyma17g37430](http://chibba.pgml.uga.edu/duplication/index/locus_app?lc=Glyma17g37430) | D2 | 41190244-41191603 |  | satt256 | 40792477-40792710 | 397.53 | BS |
| AT1G27760 (IFRD) | interferon-related developmental regulator family protein/ IFRD protein family |  | [Glyma17g35340](http://chibba.pgml.uga.edu/duplication/index/locus_app?lc=Glyma17g35340) | D2 | 39300063-39294671 |  | satt413 | 40042651-40042960 | 742.59 | BS |
|  |  |  |  |  |  |  | satt672 | 40120100-40120350 | 820.04 | LH ; FWR; BS |

LR: Length of main root; FWR: Fresh weight of root; DWR: Dried weight of root; LH; Length of hypocotyl; BS: Biomass
