# Supplementary material for: Epistatic Association Mapping for Alkaline and Salinity Tolerance Traits in the Soybean Germination Stage
Source: PLoS One. 2014 Jan 8;9(1):e84750. doi: 10.1371/journal.pone.0084750 (PMC3885605; doi:10.1371/journal.pone.0084750)
Supplement: Table S3 — SSR markers located near salt tolerance genes in soybean. (DOC) [file pone.0084750.s003.doc]

**Table S3**. SSR markers located near salt tolerance genes in soybean

| **Salt tolerance genes in soybean** | | | |  | **SSR markers that are around soybean homologous gene** | | | |
| --- | --- | --- | --- | --- | --- | --- | --- | --- |
| **Gene** | **Function** | **Chr** | **Position** |  | **Marker** | **Position** | **Distance of marker and gene (bp)** | **Trait** |
| LOC100808889 | Probable salt tolerance like protein At1g78600 like | K | 17018059-17021616 |  | satt417 | 17539835-17540158 | 518.22 | LR-STI; FWR-ATI; DWR –ATI; BS-ATI |
| LOC100807827 | probable salt tolerance like protein At1g75540 like | D2 | 41190191-41191724 |  | satt256 | 40792477-40792714 | 397.48 | BS |
| LOC100800981 | probable salt tolerance-like protein At1g75540-like | C1 | 692631-696694 |  | satt565 | 511086-511274 | 181.36 | BS; LR-STI |
| LOC100795117 | probable salt tolerance-like protein At1g78600-like | B1 | 5584068-5587113 |  | satt509 | 6206850-6207084 | 619.74 | FWR; LR-STI; DWR –ATI |
|  |  |  |  |  | sat_261 | 5835643- 5835906 | 248.53 | DWR -ATI |
| LOC100814727 | salt tolerance protein-like | F | 42123062-42125770 |  | satt656 | 41884911-41885065 | 238.00 | BS |
| LOC100797515 | salt tolerance protein-like | E | 2374803-2381861 |  | satt411 | 2517275-2517372 | 135.41 | LH-STI |

LR: Length of main root; FWR: Fresh weight of root; DWR: Dried weight of root; LH; Length of hypocotyl; BS: Biomass
